# Effectiveness of a short-term multimodal prehabilitation program in adult patients awaiting selective cardiac surgery: study protocol for an open-label, pilot, randomized controlled trial

**DOI:** 10.3389/fcvm.2023.1201737

**Published:** 2023-06-30

**Authors:** Wen Gao, Hongyan Li, Yuaner Chen, Yuping Zhang, Mao Zhang, Jingfen Jin

**Affiliations:** ^1^ Nursing Department, The Second Affiliated Hospital of Zhejiang University School of Medicine, Hangzhou, China; ^2^ Emergency Department, The Second Affiliated Hospital of Zhejiang University School of Medicine, Hangzhou, China; ^3^ Department of Rehabilitation, The Second Affiliated Hospital of Zhejiang University School of Medicine, Hangzhou, China; ^4^ Department of Cardiovascular Surgery, The Second Affiliated Hospital of Zhejiang University School of Medicine, Hangzhou, China; ^5^ Key Laboratory of the Diagnosis and Treatment of Severe Trauma and Burn of Zhejiang Province, Zhejiang University, Hangzhou, China

**Keywords:** prehabilitation, cardiac surgery, exercise, nutrition, mindfulness

## Abstract

**Background:**

Prehabilitation has been demonstrated to positively impact postoperative recovery in patients undergoing selective cardiac surgery. However, the optimal modules included in prehabilitation programs are yet to be fully explored, as existing studies have primarily focused on exercise. This study will explore the effectiveness of a three-arm prehabilitation program among adult patients awaiting selective cardiac surgery.

**Methods and analysis:**

A single-center, parallel-group randomized controlled trial will be conducted at the Second Affiliated Hospital of Zhejiang University School of Medicine (SAHZU). A total of 152 adult patients scheduled for elective cardiac surgery (coronary artery bypass grafting or valvular surgery) will be recruited from a tertiary teaching hospital. The patients will be randomly assigned to either the control group or the prehabilitation group. Patients assigned to the control group will receive standard care, which includes patient education and counseling as well as personal guidance on exercise, breathing, and coughing. Patients in the intervention group will be provided a multimodal prehabilitation program, including nutrition guidance, a diet journal, mindfulness training, and exercise guidance. The interventions will begin with home-based training and continue after hospital admission and before surgery. The primary outcome will be the perioperative 6-minute walk distance (6 MWD). The secondary outcomes will include preoperative readiness, postoperative recovery, and patient experience with the program.

**Discussion:**

The purpose of the study is to examine whether a short-term multimodal prehabilitation program will be associated with improved preoperative readiness and postoperative outcomes. The findings of this study will provide evidence to support the development of a perioperative program aimed at enhancing patient recovery.

**Clinical Trial Registration:**

www.ClinicalTrials.gov; identifier: NCT05503004.

## 1. Introduction

Valve surgery and coronary artery bypass grafting (CABG) are the most common types of cardiac surgery in China, accounting for 49.2% of all heart surgeries performed in 2021 (1). However, these surgeries can potentially lead to cognitive and functional decline (2, 3), resulting in reduced independence and a diminished health-related quality of life (HRQOL) (4). Therefore, it is of paramount importance to enhance the physical and psychological reserve of patients in order to improve their readiness for surgical stress, ultimately promoting patient recovery.

Prehabilitation refers to the process of enhancing an individual's functional capacity in preparation for upcoming surgery (5). The current guidelines of the Enhanced Recovery Association recommend that prehabilitation should be incorporated into perioperative care for cardiac surgery (6). As the first step in the enhanced recovery process, prehabilitation can mitigate sympathetic nervous overreaction and enhance patients' physical and psychological resilience (7, 8). Although studies have indicated that multimodal, multidisciplinary preoperative interventions are more effective than single-exercise interventions (9), the content of preoperative interventions prior to cardiac surgery has been primarily limited to exercises, such as respiratory muscle training and aerobic training (10). Another important consideration is the duration of prehabilitation, which has ranged from five days to ten weeks (11). Additionally, the suitability and scheduling of prehabilitation programs could significantly affect patient participation and adherence (12). Given the shortened waiting duration before hospital admission, there is a need for a short-term prehabilitation program to enhance preoperative recovery.

Recent research has provided evidence indicating the importance of preoperative nutritional evaluations and psychological interventions. It has been observed that between 12%–42% of patients undergoing cardiac surgery suffer from malnutrition before surgery, which can increase the inflammatory response and negatively impact postoperative recovery (13). Several standardized scoring systems have been developed to assess the preoperative nutritional status of patients (14), but routine risk screening for malnutrition is not currently incorporated into prehabilitation programs, and optimal methods for providing preoperative guidance are overlooked. In addition to physical and nutritional status, psychological distress can also initiate a stress response prior to surgery and dysregulate postoperative immune function, leading to worse outcomes (15). Mindfulness-based interventions have been found to have favorable effects on psychological outcomes. A study conducted on patients with heart disease found that mindfulness can decrease heart rate and improve exercise capacity (16). Furthermore, evidence indicates that mindfulness-based intervention can reduce preoperative emotional distress and lead to less emotional pain perception after surgery (17). Therefore, mindfulness may be a valuable addition to prehabilitation program, and further research exploring the use of mindfulness in this context is needed.

Additionally, it is important to note that the three prehabilitation modules – exercise, nutrition, and psychological support – can also have an interdependent relationship. Qualitative interviews with patients who underwent elective cardiac surgery revealed that physical factors such as reduced mobility and weakness can easily lead to negative emotions in patients, which may hinder their participation in rehabilitation programs (18). Psychological distress and malnutrition have also been identified as potential barriers to participation in rehabilitation (19). Conversely, exercise has been shown to improve emotional distress such as anxiety and depression in patients with heart disease by reducing stress and behavioral activation (20). This suggests that the modules in prehabilitation programs can interact with each other and may have a synergistic effect.

Therefore, the aim of this study is to implement a short-term multimodal prehabilitation program and investigate the effectiveness of such a program in improving physical function in patients receiving selective cardiac surgery. Additionally, secondary outcomes will be assessed, including preoperative readiness (preoperative anxiety, frailty, and preoperative nutritional risks), postoperative recovery (delirium, frailty, HRQOL, and length of stay), and patient experience with the program.

## 2. Methods

### 2.1. Trial design

**Figure 1:**
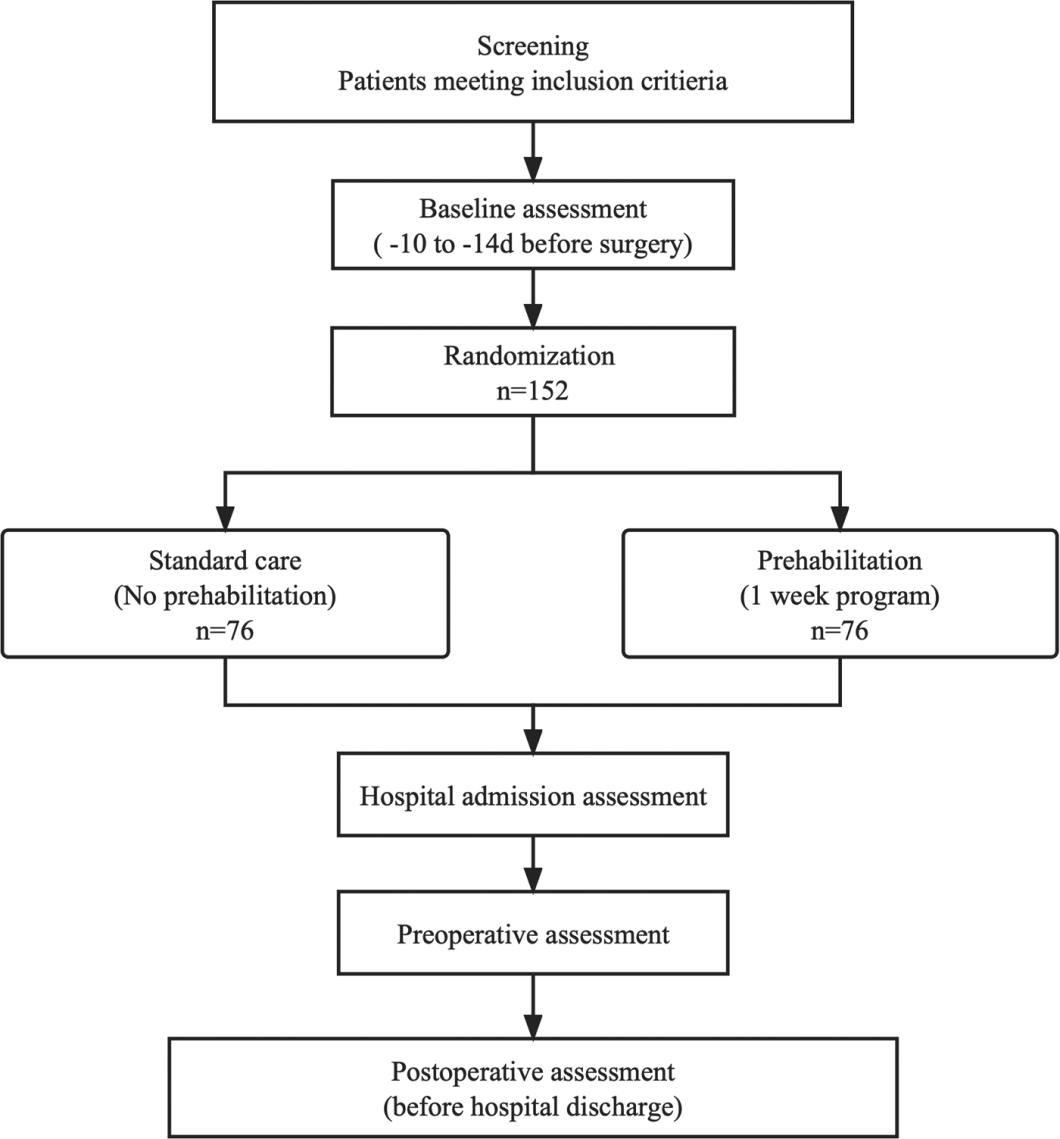
Study flowchart.

This study is a single-center, prospective, parallel group, randomized clinical trial with an allocation ratio of 1:1, comparing a one-week prehabilitation program to usual care at the Second Affiliated Hospital of Zhejiang University (SAHZU), located in Hangzhou, China. The overall trial design is illustrated in Figure 1. The trial protocol was developed following the Standard Protocol Items: Recommendations for Interventional Trials (SPIRIT) guidelines (21) and was pre-registered at clinicaltrials.gov (NCT05503004).

### 2.2. Study setting

This trial will be conducted at a tertiary academic hospital in Hangzhou, China (SAHZU). Patients will be assessed for eligibility at their initial visit to the cardiovascular clinic after receiving a clinical diagnosis requiring selective open-heart surgery. The one-week interventions will be performed at the patient's home while awaiting hospital admission and after hospital admission before surgery.

### 2.3. Eligibility criteria

Patients will be eligible for participation if they are 18 years or older and are referred for selective cardiac surgery with an expected waiting time of 7 days prior to the surgical procedure. The exclusion criteria will be as follows: (1) comorbid medical, physical, and mental conditions that contraindicated exercise or mindfulness; (2) acute or unstable cardiac conditions (e.g., unstable angina or symptomatic severe aortic stenosis); (3) disabling orthopedic and neuromuscular disease; hearing disorder; (4) dementia; (5) cardiac failure (New York Heart Association functional classes III and IV); (6) hospitalization or mechanical ventilation in the past 30 days; and (7) previous history of cardiac surgery. Patients will be withdrawn from the trial if: (1) they request to do so without any reasons; (2) they develop adverse events due to prehabilitation (e.g., fall); (3) they are referred to the transcatheter cardiac surgery after hospital admission.

### 2.4. Interventions

#### 2.4.1. Prehabilitation group

Patients assigned to the prehabilitation group will receive a prehabilitation program in conjunction with their usual preoperative care. The program will be provided by a multidisciplinary team consisting of physicians, rehabilitation therapists, nurses, and nutritionists. Patients will engage in a structured one-week prehabilitation program starting from their entry into the waiting list before selective surgery, both at home and after their hospital admission.

EXERCISE module will be composed of two sessions, namely aerobic exercise and resistance training. Aerobic exercise will require patients to walk or engage in stationary cycling for 30 min, which includes a 5-minute warm-up and cool-down activity at least three times per week. Resistance training, on the other hand, will use elastic bands for the upper and lower limbs, chest, and core muscles with ten repetitions per major muscle group for three days. Videos demonstrating the exercise routines will be sent to the mobile phones of patients and their accompanying family members during hospitalization. The intensity of each patient's exercise will be personalized based on their health status, exercise performance, and training response. The appropriate heart rate ranged will be within the range of (220 – age) × (50%–60%) and we will monitor the patient's heart rate and subjective perceived exertion [Borg rating of perceived exertion (RPE)] throughout the exercise using a wristband (Xiaomi Wristband 7, Xiaomi Technology Co., Ltd.).

NUTRITION interventions will consist of early screening for malnutrition and structured education to reduce fat-rich diets and increase high-quality protein uptake. Patients will be encouraged to consume healthy, high-protein diets including high-quality protein sources such as eggs, fish, lean meats, or dairy. The optimal suggested diet goals will be set at 1.5–2 g/kg/d of protein from routine food intake. To assess the daily protein intake, patients will be recommended to keep diet journals, record and take photos of their three daily meals, which will then be evaluated and guided by a nutritionist. Malnourished patients will be transferred to a face-to-face nutrition clinic, and high-protein oral nutritional supplement (ONS) servings will be provided daily.

PSYCHOLOGY intervention will involve a one-week brief mindfulness practice session, with 10 min of practice per day before sleep. This intervention will focus on breathing mindfulness and feelings, guided by an audio-mindfulness dialogue. Psychologists will introduce a short mindful breathing practice after patient allocation. Mindfulness guidance recordings will be sent to the patient's mobile phone, and the practice of mindfulness on breathing and feeling will be encouraged.

#### 2.4.2. Control group

Patients assigned to the control group will not receive any additional prehabilitation intervention. Instead, they will receive usual care, which will include a thorough admission assessment, education on inspiratory muscle training, breath exercises, coughing, online health education, counselling, and nutrition risk assessment.

### 2.5. Outcomes

The timeline for this study will be one week. Prehabilitation will be implemented one week before the surgery, and the follow-up period will comprise of the perioperative period until the patient's discharge from the hospital. The outcomes will be assessed at four different time points: before the intervention (T0), upon admission to the hospital (T1), before surgery (T2), and after surgery (T3, before hospital discharge). Table 1 shows the complete assessment timeline.

**Table 1:** Overview of assessment.

Measurement	T0: baseline	T1: hospital admission	T2: 1 day before surgery	T3: after surgery (before hospital discharge)
6-minute walk distance (6MWD)	×	×	×	×
Preoperative anxiety (APAIS)	×	×	×	
Frailty (FFPC)	×		×	×
Nutrition risks (NRS2002)	×	×		
Prehabilitation experience (FSS)		×	×	
ICU delirium (CAM-ICU-7)				×
Postoperative delirium (4AT)				×
Health-related quality of life (EQ-5D-5l)	×	×	×	×
Length of ICU stay, days				×
Length of hospital stay, days				×
Length of mechanical ventilation, hours				×

**The primary outcome** was the change in functional capacity over time, measured as the difference in absolute change in 6-minute walk distance (6 MWD) between T0 and T2 (primary analysis), as well as between T0 and T3. In this regard, a change of 20 meters from the baseline was deemed significant, indicating either an improvement or deterioration. To conduct this evaluation, participants will be instructed to wear comfortable footwear and walk back and forth in a 20-meter stretch of hallway for 6 min, aiming to reach a point of exhaustion by the end of the exercise. The tests will be conducted under the supervision of a blinded assessor following a standardized procedure to minimize potential sources of error due to bias or different levels of encouragement.

**Secondary outcomes** will comprise preoperative readiness (preoperative anxiety, frailty, and preoperative nutritional risks), postoperative recovery (delirium, frailty, HRQOL, and length of hospital stay), and patient experience with the program. Preoperative anxiety will be evaluated based on the Amsterdam Preoperative Anxiety and Information Scale (APAIS), perioperative frailty will be assessed using the Fried Frailty Phenotype Criteria (FFPC), and preoperative nutritional risk will be screened using nutritional risk screening (NRS 2002). The Flow State Scale short version will be used to examine patient experience of prehabilitation. Delirium will be assessed using the Confusion Assessment Method of the Intensive Care Unit -7 (CAM-ICU-7) and the 4AT Rapid clinical test for delirium for every nurse shift. HRQOL will be assessed using the EuroQol 5 Dimension 5 Level (EQ-5D-5l). The postoperative lengths of ICU stay, hospital stay, and mechanical ventilation will be recorded.

### 2.6. Recruitment

Individuals who meet the eligibility criteria will be presented with a comprehensive overview of the study's aims and procedures by a cardiac rehabilitation therapist if they express an interest in participating. The initial appointment will be scheduled 10–14 days before their hospital admission. Written informed consent will be collected from all participants at the hospital preparation center before the completion of the study assessments.

### 2.7. Randomization and blinding

Eligible patients will have an equal opportunity to be randomly assigned to either the prehabilitation group or the control group in a 1:1 ratio. Randomization will be performed via a computer-generated block scheme and the resulting group assignments will be placed in opaque envelopes with sequential numbering by an independent research assistant not involved in this study. The research assistant will open a concealed envelope to allocate patients to their respective group. The main interventionist, assessor, and statistician will remain unaware of the group assignments until the envelope is sealed. Due to the intervention's inherent nature, it will not be possible to mask the participants or healthcare professionals.

### 2.8. Data collection and management

At the baseline interview after randomization, demographic and clinical characteristics will be collected, including age, sex, BMI, ASA physical status class, comorbidities such as diabetes, hypertension, and hyperlipidemia, and Charlson Comorbidity index. Laboratory test results and assessment outcomes will be obtained from patients' electronic medical records, including serum hemoglobin, NRS 2002, HYHA, main diagnosis, and EuroScore. Additionally, data on daily exercise amount and intensity, diet details, and duration of mindfulness will be collected using a bracelet (Xiaomi Wristband 7, Xiaomi Technology Co., Ltd.). The 6 MWD test will be performed and recorded by trained researchers at our institution after providing standardized instructions and demonstrations of the tests. Patient-reported questionnaires will be administered, and the outcomes will be recorded by trained researchers. During the prehabilitation period, message interviews will be sent to patients every day, and feedback on their daily prehabilitation performance will be documented. After the surgery, information on the surgery, duration of ICU stay, and postoperative recovery will be obtained from electronic records. Patient identity and personal information will remain confidential unless consent is provided. All collected data will initially be recorded in a paper-based case report form and then entered into a digital database by an independent investigator.

### 2.9. Sample size

We determined that a difference of 20 meters in the change in 6-minute walk distance (6 MWD) between T0 and the T2 (primary analysis) in the prehabilitation group compared to the control group was a meaningful clinical outcome based on a previous study (22). Consequently, we conducted a sample size calculation using G*Power (version 3.1.) for the repeated-measures ANOVA with an alpha error of 5% and power of 0.95, with two groups and four measurements. The estimated sample size required was 132 patients. To account for a 15% attrition rate during the intervention period, the target sample size was increased to 152 participants (76 per group).

### 2.10. Statistical analysis

To compare baseline characteristics between groups, we will use an independent-samples t-test for continuous variables and a *χ*^2^ Test for categorical variables. To analyze differences in primary outcomes, we will use repeated-measures analysis of variance. All statistical analyses will be conducted using SPSS (version 27.0; IBM) and R (version 4.0.5).

## 3. Discussion

Although enhanced recovery has been recommended and practiced for years in cardiac surgical patients, prehabilitation before surgery has not received sufficient attention. Due to impaired cardiopulmonary function and limited daily activities, patients are at risk of functional deterioration while waiting, which could lead to surgical delay and form a vicious circle (22). Another important consideration is the waiting time and duration of prehabilitation. A study reported that a delay of more than 7 days in patients requiring CABG was associated with worse in-hospital and long-term outcomes (23). Therefore, there is a need for short-term prehabilitation programs to quickly prepare patients for surgery. Further research is required in this field to better understand the clinical effectiveness of a short-term prehabilitation programs before surgery and to combine home and hospital prehabilitation as an effective bridge to achieve preoperative readiness.

The aim of this study is to investigate the impact of the prehabilitation on preoperative readiness and postoperative recovery. To date, while studies have mainly focused on postoperative functional performance and recovery (10), there is increasing evidence that preoperative readiness is essential for successful postoperative recovery. In particular, patient readiness for surgery has been associated with shorter mechanical intubation times and reduced length of stay after CABG (24). Preoperative frailty is also an important indicator in preoperative decision-making and can help predict patient recovery trajectory (25). It is also worth noting that patients undergoing cardiac surgery often experience high levels of preoperative anxiety (26), highlighting the importance of psychological interventions to improve patients' preoperative psychological readiness. In addition, malnutrition has been linked to a longer postoperative recovery time and higher risk of cardiovascular and infectious complications (13), indicating the need for a multimodal prehabilitation that includes elements to improve physical, psychological, and nutritional factors. To address these needs, we have designed a three-arm program that incorporates exercise, nutritional optimization, and mindfulness.

This study will implement a comprehensive prehabilitation program that will be both home- and hospital-based, covering the entire waiting period before surgery. Our proposed prehabilitation program is designed to optimize preoperative and postoperative physical function performance and improve psychological well-being by enhancing physical function and alleviating psychological distress. This program was designed and will be supervised by physiotherapists, psychologists, nutritionists, cardiac rehabilitation nurse specialists, and cardiac surgical physicians, and the intensity of the program will be tailored to each patient's needs to ensure safety and effectiveness both practiced at home and in hospital. If this program is proven effective, it could provide significant benefits to patients on the waiting list by offering home-based prehabilitation and continuing care programs after hospital admission and postoperative recovery.

This study has some limitations. First, the study only includes patients awaiting for CABG and/or valve surgery. Second, this is a single-center study. Therefore, further multicenter studies including more patients with various diagnoses are needed. Third, due to the time window of 7 days, although the nutritional intervention may be beneficial for postoperative recovery, measurable changes in body composition or serum albumin concentrations may not be detectable.
